# SYMptoms in chronic heart failure imPACT on burden of treatment (SYMPACT): a cross‐sectional survey

**DOI:** 10.1002/ehf2.13904

**Published:** 2022-04-21

**Authors:** Rosalynn C. Austin, Lisette Schoonhoven, Vasiliki Koutra, Alison Richardson, Paul R. Kalra, Carl R. May

**Affiliations:** ^1^ School of Health Sciences, Faculty of Environmental and Life Sciences University of Southampton Southampton UK; ^2^ Department of Cardiology Portsmouth Hospitals University NHS Trust (PHU) Queen Alexandra Hospital, Cardiology Research Nurses, C‐Level, Southwick Hill, Cosham Portsmouth PO6 3LY UK; ^3^ National Institute for Health Research (NIHR) Applied Research Collaboration (ARC) Wessex Southampton UK; ^4^ Julius Center for Health Sciences and Primary Care, University Medical Center Utrecht Utrecht University Utrecht The Netherlands; ^5^ Centre for Sport, Exercise and Osteoarthritis Research Versus Arthritis Southampton UK; ^6^ University Hospital Southampton NHS Foundation Trust, Southampton General Hospital Southampton UK; ^7^ Institute of Health and Wellbeing, College of Medical, Veterinary and Life Sciences University of Glasgow Glasgow UK; ^8^ Faculty of Science and Health University of Portsmouth Portsmouth UK; ^9^ Faculty of Public Health and Policy London School of Hygiene and Tropical Medicine London UK; ^10^ Applied Research Collaboration (ARC) North Thames National Institute for Health Research (NIHR) London UK

**Keywords:** Chronic heart failure, Burden of treatment, Symptoms, Cross‐sectional survey, Self‐care

## Abstract

**Aims:**

This study aimed to describe patient‐reported symptoms and burden of treatment (BoT) experienced by patients with chronic heart failure (CHF). BoT describes the illness workload, individual capacity to perform that work, and resultant impact on the individual. Overwhelming BoT is related to poor quality of life and worse clinical outcomes. This research is the first to explore symptoms and BoT in people with CHF, in the UK.

**Methods and results:**

This is a cross‐sectional questionnaire survey of CHF patients. Participants completed the Heart Failure Symptom Survey (HFSS; max score 10) and the Minnesota Living with Heart Failure Questionnaire (MLHFQ; max scores: physical 40, emotional 25, and total 105), which measured symptoms. BoT was measured with the Patient Experience with Treatment and Self‐management (PETS; max score 100) questionnaires. Participant characteristics and questionnaire results were summarized using descriptive statistics. Relationships between symptoms and BoT, summarized by the workload and impact indices, were explored using Spearman's and Pearson's correlation coefficients together with scatter plots. The survey was completed by 333 participants, mean age of 71 (±13) years old. The majority (89%) were recruited from secondary care NHS trusts, and 25% were female. All types of heart failure were represented. Mean symptom scores were as follows: HFSS burden score: 2.4 (±2.1), and MLHFQ scores: physical score 20 (±12.4), emotional score 9.9 (±8.1), and total score 41.3 (±26.3). The highest mean PETS domain scores were *exercise* [51.3 (±24.7)], *diet* [40.3 (±22.7)], *difficulty with healthcare services* [39.9 (±21.3)], and *physical and mental fatigue* [36.0 (±25.7)]. Pairwise correlations were observed between HFSS scores and MLHFQ physical and emotional sub‐scores with PETS workload and impact indices. Positive correlations were weak to moderate (0.326–0.487) between workload index and symptoms, and moderate to strong between impact index and symptoms (0.553–0.725). The *P* value was 0.006, adjusted by Bonferroni's correction.

**Conclusions:**

Symptoms are associated with BoT in CHF patients. Although symptom burden was low, CHF patients reported higher levels of burden around self‐care activities of exercise, diet, healthcare interaction, as well as physical and mental fatigue due to engagement with self‐care regimens. Observed higher levels of burden were in key self‐care areas for CHF and suggest areas where service delivery and support of CHF patients may be improved to reduce BoT. Clinicians could individualize their consultations by focusing on troublesome symptoms, as well as alleviating illness workload, which may better enable patients to live well with CHF.

## Introduction

While advances in treatment for chronic heart failure (CHF) have improved patient survival, symptoms of CHF can be progressively debilitating.^1^ More severe symptoms have been linked to increased risk of adverse clinical events like hospitalizations.^2^ Symptoms of HF have a negative impact on health‐related quality of life (HRQoL) and contribute to psychological distress.^1^, ^2^ According to Riegel *et al*.,^3^ theory of self‐care, monitoring, and managing symptoms are now considered part of the work patients are expected to engage with while living with a chronic illness. Self‐care refers to the self‐monitoring of illness processes, treatment and healthcare management tasks, as well as behaviour changes aimed at improving patient HRQoL and clinical outcomes. Self‐care is a central component of CHF care^4^, ^5^ and a key component of a patient's burden of treatment (BoT). The theory of BoT describes the dynamic interaction between an individual's capacity (capability and resources), workload assigned by healthcare professionals (treatments and self‐care regimens), together with the resultant impact on that individual's life.^6^, ^7^, ^8^, ^9^ Similar to the theory of self‐care, the theory of BoT acknowledges symptoms as part of the work but does not specify the influence that CHF symptoms like breathlessness or fatigue might have on self‐care engagement or on experienced BoT.

In chronic illness, including CHF, overwhelming BoT is thought to contribute to poor adherence to self‐care regimens.^9^, ^10^ Failure of CHF patients to engage with self‐care activities is not well understood but considered a common reason for recurring admissions and poor outcomes.^11^ BoT specific to CHF has been linked to multiple medications and disjointed healthcare services.^12^, ^13^ Emotional burden and an individual's capacity have been highlighted as factors that increase BoT in CHF.^14^ Physical symptoms are thought to contribute to emotional burden.^15^ Heart failure symptoms (e.g. fatigue and breathlessness) could understandably make tasks like attending hospital appointments much harder and increase BoT. The relationship between symptoms of CHF and BoT has not been explored.

### Objectives

The aim of this study was to explore the interaction between symptoms and BoT in CHF, with the hypothesis that higher reported symptoms relate to higher scores of BoT.

## Methods

### Design

The SYMPACT study design was previously published.^16^ This paper reports on Phase I, an observational cross‐sectional survey of CHF patients' symptoms and BoT measured by validated questionnaires. Adults with CHF in three NHS trusts in Hampshire, UK (secondary and primary care services), in receipt of inpatient and outpatient care or a member of a community support group were invited to participate. Individuals were given the choice to complete the three questionnaires with the support of research staff or to complete them independently, returning questionnaires and consent by post. Written full informed consent was received from all participants.

Inclusion criteria were English‐speaking adults over the age of 18. Participants with a clinical diagnosis of heart failure (for at least 6 months), with a broad range of New York Heart Association (NYHA) classification and left ventricular ejection fractions, who were prescribed a minimum of one treatment for heart failure were considered. The exclusion criteria were patients with or waiting for heart transplants, receiving palliative care, or with substantial cognitive impairment (in the investigator's opinion).

### Data collection

Personal characteristics of participants (age, gender, height, weight, ethnicity, marital status, and living situation), clinical information (co‐morbidities, medications, and CHF diagnosis date), and CHF details (CHF type, aetiology, NYHA classification, left ventricular ejection fraction, and oedema level) were collected from participants and their medical records. Participants completed three validated and reliable questionnaires.

#### Heart Failure Symptom Survey

The Heart Failure Symptom Survey (HFSS) is a disease‐specific tool that measures 14 CHF symptoms (e.g. breathlessness, fatigue, and oedema). Each symptom is measured on a Likert scale (from 0 to 10) where a higher score represents a more severe experience. Each symptom is captured in four dimensions: symptom frequency and severity, as well as interference with physical activity and enjoyment of life.^17^ HFSS includes both typical and atypical symptoms of CHF.^18^ HFSS burden score (max score 10) was calculated. This is a standardized summation of two dimensions (frequency and severity) of all 14 symptoms, quantifying the reported burden of symptoms. Individual symptom scores (max score 10) were also calculated as a standardized summation of all four dimensions for each symptom. A score of <1 was chosen to indicate that the participant denied experiencing that symptom, as it meant that the participant did not report individual items as high enough to result in a summed total score of >1. Participants were asked to report on their symptoms from the last week.

#### Minnesota Living with Heart Failure Questionnaire

The Minnesota Living with Heart Failure Questionnaire (MLHFQ) is a tool that measures key physical, emotional, social, and mental dimensions of HRQoL.^19^ The MLHFQ physical and emotional sub‐scores were calculated^20^ and used to describe participants' symptoms. Additionally, the MLHRQ total score was calculated. Maximum MLHFQ total score is 105, and the sub‐scores have maximum scores of 40 (physical) and 25 (emotional). Higher scores are related to worse HRQoL or symptom experience. Participants were asked to report on their experience over the last month.

#### Patient Experience with Treatment and Self‐management

The Patient Experience with Treatment and Self‐management (PETS) questionnaire is a tool that quantifies the patient's experience of BoT. It was designed for use in multi‐morbid chronic illness.^21^ While the 48‐item tool was used, we report results using the more recently revised Brief PETS.^22^ The Brief PETS is a 34‐item tool that has reduced the original nine domains into two indices and four individual domains. The workload index summarizes the domains of medical information, medications, medication appointments, and monitoring health. The impact index summarizes the domains of role and social activity limitations together with physical/mental fatigue. The four individual domains are diet, exercise/physical therapy, medical expenses, and difficulty with healthcare services.^22^ Maximum score of the indices or domains is 100, with higher scores relating to greater burden. In the domains of medical information, monitoring health, medical expenses, and difficulty with healthcare services, the PETS scoring system requires the participant response ‘did not apply’ to be converted into missing data. In the domains of diet and exercise/physical therapy, participants are asked a yes/no question where ‘no’ means they do not continue with the items in this domain. Both data processing techniques increase the amount of missing data. It also provides the opportunity to describe how many participants reported not having diet of exercise/physical therapy discussed with them by a healthcare provider. As PETS does not have a single summary score, indices (workload and impact indices) were used as summative measures for BoT and used in the correlation analysis. Participants were asked to report on their experience over the last month.

### Data analysis

Statistical analyses were performed using the Statistical Package for Social Sciences (SPSS) Version 26.^23^ Descriptive statistics were performed on participants' clinical data and questionnaire responses. Data were summarized as mean (±standard deviation) and median [with range and inter‐quartile range]. Correlations and scatter plots were obtained to assess the hypothesized pairwise relationships between symptoms (HFSS burden score and MLHFQ physical and emotional sub‐scores) and BoT (PETS workload and impact indices). The assumption of normality was assessed using the Kolmogorov–Smirnov and Shapiro–Wilk normality tests and visually examined using histograms and normal Q–Q plots. Results with *P* value less than 0.05 were considered statistically significant, and Bonferroni method was used when required to adjust for multiple testing. Correlation coefficient above 0.5 was considered at least moderate.^24^

### Ethics

Ethical approval was granted by University of Southampton Ethics Committee (ERGO: 41287) and the Nottingham HRA1 Research Ethic Committee, Health Research Authority (MREC: 18/EM/0339, IRAS: 247773). SYMPACT conforms with the Declaration of Helsinki principles.^25^ SYMPACT was registered with ISRCTN registry: ISRCTN11011943.

### Procedures

Screening of participants occurred between November 2018 and March 2020. They were approached by a research team member while attending NHS trusts (inpatients and outpatients) or community support groups. After providing time for participants to consider participation, fully informed written consent was received either in person or through the post. Method of consent was chosen by participants.

Participants who completed the study activity in an NHS trust were offered the support of a research nurse, to answer specific queries regarding the questionnaires. Participants who chose to self‐consent were asked to return the completed questionnaires and consent form by post in provided self‐addressed stamped envelopes. If the study packs were not returned, participants were called, and if interest in participation was confirmed, then participants were reminded to complete and return the study packs to the appropriate study centre. Clinical data from medical records were collected by research staff. These data along with the completed questionnaires: HFSS,^17^ MLHFQ,^19^ and PETS,^21^ were entered into an electronic database. All data were managed by REDCap Cloud software.^26^ All data entered in the database by research staff at local centres were verified by a second researcher. Identified errors, mainly typographical, were corrected.

## Results

Adults with CHF (*n* = 633) were screened across three NHS trusts and community support groups in Hampshire, UK: 387 were eligible and approached to participate, 338 consented, and 333 completed study activities. Those found not to be eligible included individuals with new diagnosis of CHF, receiving end of life care, or with cognitive concerns. Those who chose not to participate mainly cited inconvenience of the study for non‐participation.

### Survey characteristics

Most participants (89%) were recruited from secondary NHS trusts with the remainder of participants from primary care trusts (9%) or community support groups (2%). A minority of participants (26%, *n* = 88) reported receiving help from carer/family member to complete the questionnaires. Only four questionnaires (three HFSS and one MLHFQ) were not completed or were missing in study packs returned by post.

### Study population

Participants had an average age of 71 (±13 years) with an age range of 22–96 years old. The majority or participants were male (72%) and identified as White British (79%). Over half of the patients reported living with a spouse or partner (58%). Detailed study population characteristics are presented in *Table* *1*.

**Table 1:** Participant characteristics

Age (years)	
Mean (SD)	71 (13)
Missing (*n*)	1

BMI	
Mean (SD)	30.6 (6.5)
Missing (*n*)	62

Number of medications	
Mean (SD)	9 (3.3)

Number of health issues	
Mean (SD)	7 (3.4)

Co‐morbidities	*n* (%)
Atrial fibrillation	147 (44)
Myocardial infarction	67 (20)
Blood pressure disease	133 (40)
Stroke or mini‐stroke	33 (10)
Peripheral vascular disease	13 (4)
COPD	51 (15)
Diabetes (types I and II)	100 (30)
Chronic kidney disease	61 (18)
Muscle skeletal disease	83 (25)
Mental health diagnosis	22 (7)
Cancer	26 (8)

Ethnicity	*n* (%)
White British	263 (79)
White other	6 (1.8)
Asian	3 (0.9)
White and Asian	1 (0.3)
Missing	60 (18)

Marital status	*n* (%)
Married	186 (55.9)
Civil partnership	11 (3.3)
Divorced	36 (10.8)
Separated	5 (1.5)
Widow	54 (16.2)
Single	31 (9.3)
Missing	10 (3.0)

Living situation	*n* (%)
With partner/spouse	194 (58.3)
With other family members	31 (9.3)
On my own	93 (27.9)
Sheltered accommodation	4 (1.2)
With lodgers	2 (0.6)
Missing	9 (2.7)

BMI, body mass index; COPD, chronic obstructive pulmonary disorder; *n*, number of participants; SD, standard deviation.

Missing values include unreported by participant, not in the medical records in the past 4 months, or records not accessed.

### Chronic heart failure characteristics and treatments

All participants had a diagnosis of CHF for a minimum of 6 months. Sixteen per cent of participants were classed as heart failure with preserved ejection fraction (HFpEF) in clinical records. A third had ischaemic heart disease documented as CHF aetiology. Over half of the population (53%) had no documented NYHA classification in their medical records over the preceding 4 months. Participants were prescribed a mean of 9 (±3.3) medications and had 7 (±3.4) co‐morbidities documented. The majority of participants (66%) were on triple therapy for CHF (at least three of the medication types listed in *Table* *2*). Additionally, 72% were on some type of diuretic, and 34% had an implantable cardiac device. Further details on participant CHF characteristics and treatments are in *Table* *2*.

**Table 2:** CHF characteristics and treatments

Type of heart failure	*n* (%)
HFrEF	209 (62.8)
HFpEF	53 (15.9)
Missing	71 (21.3)

Aetiology	*n* (%)
Ischaemia	111 (33.3)
Cardiomyopathy (any type)	76 (22.8)
Hypertension	17 (5.1)
Multifactorial	16 (4.8)
Other^a^	15 (4.5)
Missing	98 (29.5)

Years since diagnosis	*n* (%)
6 months to 1 year	62 (18.6)
1–3 years	100 (30.0)
3–5 years	49 (14.7)
5–10 years	48 (14.4)
10+ years	31 (9.3)
Missing (at least 6 months)	43 (12.9)

NYHA classification	*n* (%)
I	28 (8.4)
II	71 (21.3)
III	35 (10.5)
IV	4 (1.2)
Missing	195 (58.6)

Left ventricular ejection fraction^b^	*n* (%)
Normal (≥55%)	38 (11.4)
Borderline (50–54%)	18 (5.4)
Impaired (36–49%)	94 (28.2)
Severely impaired (≤35%)	161 (48.3)
Missing	22 (6.6)

Oedema level	*n* (%)
Nil oedema	125 (37.5)
Minor (feet to mid shin)	67 (20.1)
Moderate (lower limb)	24 (7.2)
Severe (above knee)	20 (6.0)
Missing	97 (29)

CHF treatments	*n* (%)
Beta‐blocker	295 (89)
ACE inhibitor/angiotensin receptor blocker	153 (46)
Mineralocorticoid receptor antagonist	270 (81)
Sacubitril/valsartan	136 (41)
Ivabradine	23 (7)
Diuretics	239 (72)
Implantable cardiac device	124 (37)

ACE, angiotensin‐converting enzyme; CHF, chronic heart failure; HFpEF, heart failure with preserved ejection fraction; HFrEF, heart failure with reduced ejection fraction; *n*, number of participants; NYHA, New York Heart Association.

Missing values include unreported by participant, not in the medical records in the past 4 months, or records not accessed.

^a^ Other: atrial fibrillation, pulmonary hypertension, valve disease, hyperthyroidism, and rheumatic fever.

^b^ Classified according to the British Society of Echocardiography standards.^27^

### Questionnaire results

#### Heart Failure Symptom Survey

For a quarter of participants (26%), HFSS burden score was less than 1, which was interpreted as CHF symptoms created no burden (see the Methods section). Fatigue and shortness of breath with activity were most often reported to cause a degree of burden (score >1), 85% and 82%, respectively. Symptoms such as dizziness, forgetfulness, and depression were reported for at least 50% of the sample. Median HFSS burden score was 2.3 [0–9.8]. Individual symptoms scores are reported in *Table* *3*.

**Table 3:** Questionnaire results

	Mean (SD)	Median [min–max]	IQR	Missing (*n*)	Score <1 (*n*)
HFSS (*n* = 330)					
Burden score	2.4 (2.1)	2.3 [0–9.8]	3.1	3	88
Shortness of breath at rest	2.3 (3.0)	0.4 [0–10]	3.8	5	175
Shortness of breath with activity	4.7 (3.4)	4.8 [0–10]	6.3	7	63
Shortness of breath when lying down	1.6 (2.6)	0 [0–10]	2.1	3	212
Shortness of breath wake up at night	1.4 (2.4)	0 [0–10]	2.0	4	217
Swelling in lower limbs	2.4 (3.3)	0.3 [0–10]	4.5	6	183
Bloated abdomen	2.3 (3.2)	0.3 [0–10]	4.0	3	186
Fatigue	5.1 (3.5)	5 [0–10]	6.3	4	54
Chest pressure	1.7 (2.7)	0 [0–10]	2.5	5	208
Irregular heartbeat	2.0 (2.9)	0.3 [0–10]	3.5	4	186
Worsening cough	1.7 (2.8)	0 [0–10]	2.3	4	209
Dizziness	2.4 (2.9)	1 [0–10]	4.0	3	150
Difficulty sleeping	2.4 (3.2)	0.9 [0–10]	4.5	3	165
Forgetfulness	2.2 (2.9)	1 [0–10]	3.8	8	135
Depressed	2.7 (3.2)	1.3 [0–10]	4.5	9	146
MLHFQ (*n* = 332)					
Total score	41.3 (26.3)	41 [0–104]	47	1	49
Physical symptom score	20 (12.4)	21 [0–40]	22.8	1	93
Emotional score	9.9 (8.1)	9 [0–25]	15	1	175
PETS (*n* = 333)					
Workload index score	27.3 (17.1)	26.9 [0–86]	27.1	6	65
Medical information	28.8 (19.5)	25 [0–93]	28.6	9	61
Medications	20.0 (18.7)	21.4 [0–86]	28.6	1	125
Medical appointments	27.1 (22.1)	25 [0–96]	33.3	0	98
Monitoring health	33.7 (24.4)	25 [0–100]	37.5	45	56
Impact index score	33.2 (25.4)	29.6 [0–100]	40.31	7	71
Role and social activity limitations	30.4 (29.7)	25 [0–100]	45.8	2	117
Physical and mental fatigue	36.0 (25.7)	25 [0–100]	40.0	6	57
Medical expenses	31.2 (24.6)	25 [0–100]	41.25	61	68
Difficulty with healthcare services	39.9 (21.3)	38.9 [0–100]	24.04	19	36
Diet	40.3 (22.7)	33 [0–100]	22.2	195	14
Exercise/physical therapy	51.3 (24.7)	50 [0–100]	33.3	166	14

HFSS, Heat Failure Symptom Survey; IQR, inter‐quartile range; MLHFQ, Minnesota Living with Heart Failure Questionnaire; PETS, Patient Experience with Treatment and Self‐management; SD, standard deviation.

Missing values include unreported by participant or as a function of the tool scoring system (*n* = number of participants). Scores <1 are reported to illustrate the number of participants (*n*) who reported that factor as not present (HFSS and MLHFQ) or very easy (PETS).

#### Minnesota Living with Heart Failure Questionnaire

A small proportion of participants (13%) reported that CHF did not impact HRQoL (total score <1). In the symptom sub‐score, 28% (*n* = 93) denied physical symptoms and 52% (*n* = 175) denied emotional symptoms (scores <1). Symptom sub‐scores were also calculated with the mean physical score of 20 (±12.4) and emotional score of 9.9 (±8.1). The mean total MLHFQ score was 41.3 (±26.3).

#### Patient Experience with Treatment and Self‐management

In the domains *diet* and *exercise/physical therapy*, respectively, 54% and 46.5% of participants reported that no healthcare professional had given them guidance around dietary recommendations or exercise/physical therapy advice. For those who reported receiving advice in these domains, participants reported a mean score of 40.3 (±22.7) and 51.3 (±24.7), respectively. The domains of *difficultly with healthcare services* [39.9 (±21.3)] and *physical and mental fatigue* [36.0 (±25.7)] were the next most burdensome domains. Workload index had a mean score of 27.3 (±17.1), and impact index had a mean score of 33.2 (±25.4). *Table* *3* reports PETS individual domain scores.

### Correlation between symptoms and burden of treatment

**Figure 1:**
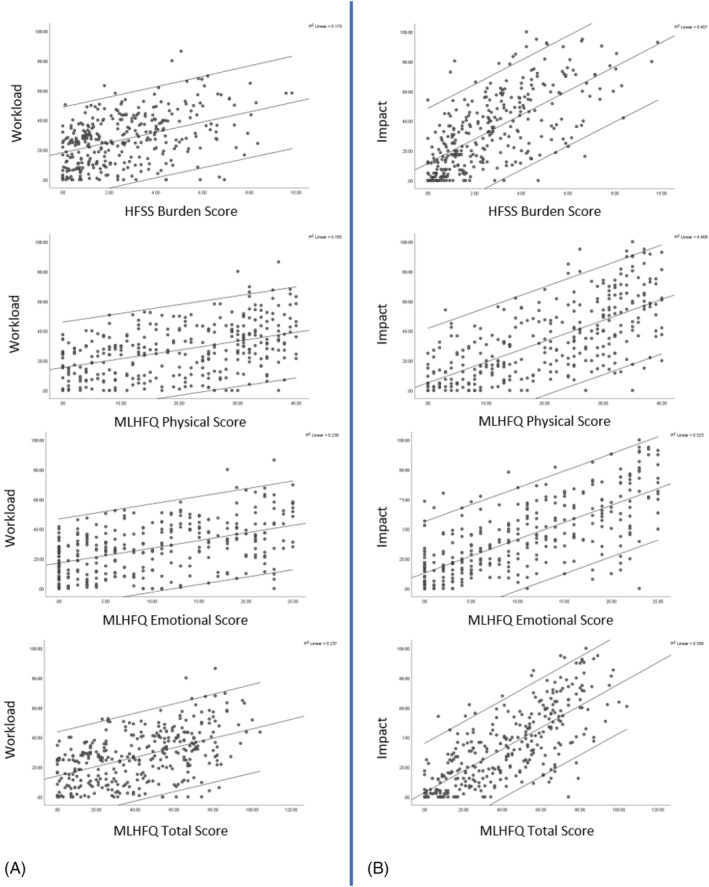
Scatter plots of burden of treatment indices [Patient Experience with Treatment and Self‐management (PETS)] and symptom scores [Heart Failure Symptom Survey (HFSS) and Minnesota Living with Heart Failure Questionnaire (MLHFQ)]. Workload is shown in (A), and impact is shown in (B). Linear line of best fit and 95% confidence lines shown.

**Figure 2:**
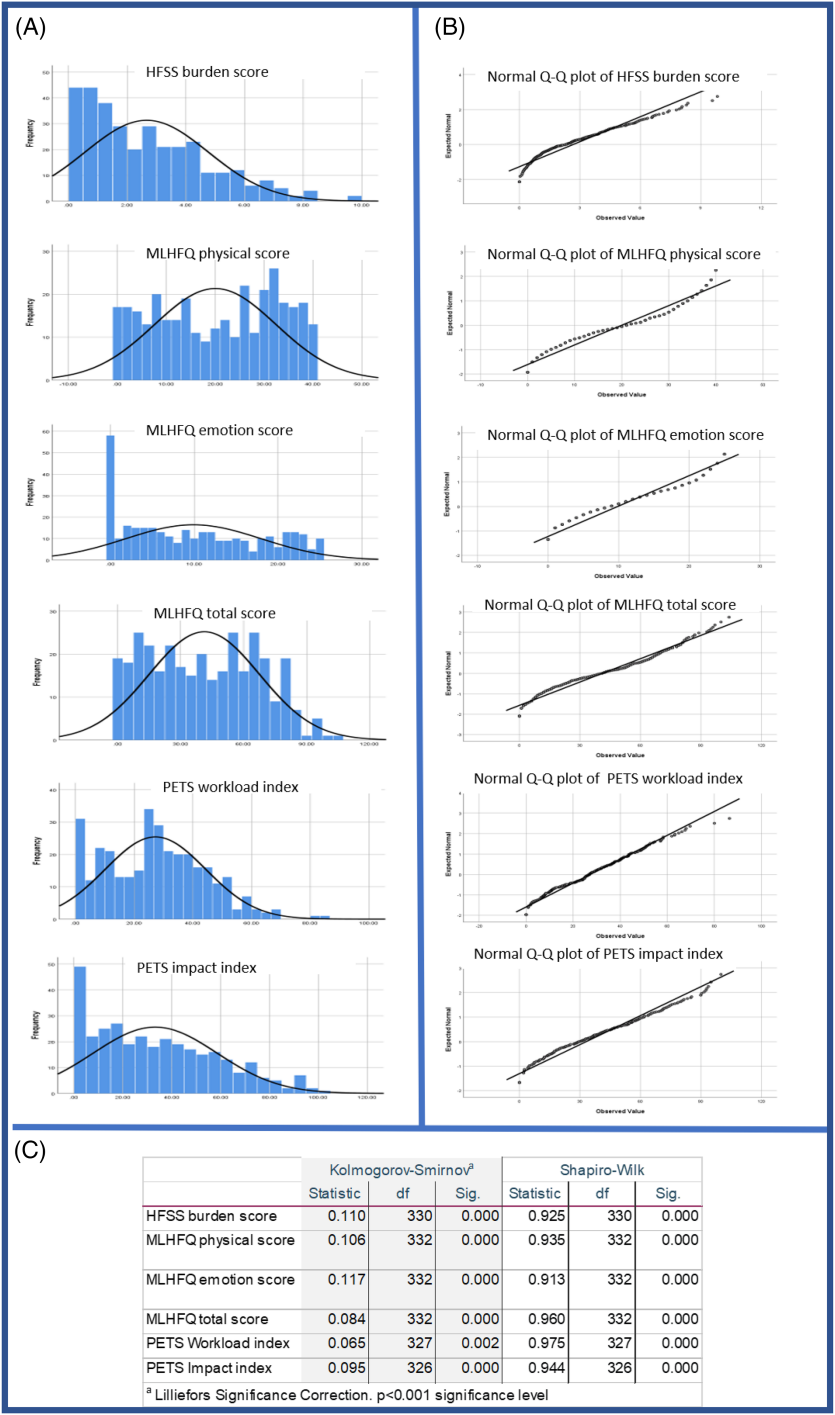
Normality examinations of Heart Failure Symptom Survey (HFSS) burden, Minnesota Living with Heart Failure Questionnaire (MLHFQ) scores, and Patient Experience with Treatment and Self‐management (PETS) indices. (A) Histograms with normal curves for all variables used in correlations. (B) Normal Q–Q curves for those same variables. (C) The normality test results.

Pairwise correlations were obtained to examine the relationship between HFSS scores and MLHFQ physical and emotional sub‐scores with PETS workload and impact indices. Scatter plots between symptom scores (HFSS burden score and MLHFQ physical and emotional scores) and BoT scores (PETS workload index and impact index) showed some indication of linear relationship (*Figure* *1*). From a combination of graphical inspection and formal testing, most scores did not follow a normal distribution except some of the MLHFW and PETS scores, where there were no strong deviations from normality according to Q–Q plots (*Figure* *2*).

Both parametric and non‐parametric correlation results are presented in *Table* *4*. All correlation coefficients are significantly different from zero using a Bonferroni correction at a 5% family‐wise significance level. Differences between the parametric and non‐parametric tests are minimal. Correlations between symptoms (HFSS burden or MLHFQ sub‐scores) and the PETS impact index score were moderate to strong (0.553–0.725). In comparison, correlations between symptoms (HFSS burden or MLHFQ sub‐scores) and the PETS workload index score were weak (0.326–0.467). Similar differences in correlation strengths were observed between HRQoL (MLFHQ total score) and the workload and impact indices.

**Table 4:** Correlations

	HFSS burden score		MLHFQ physical score		MLHFQ emotional score		MLHFQ total score	
	Correlation coefficient	*P*	Correlation coefficient	*P*	Correlation coefficient	*P*	Correlation coefficient	*P*
Workload index								
*n*	324		326		326		326	
Pearson's	0.344*	0.000	0.326*	0.000	0.406*	0.000	0.487*	0.000
Spearman's	0.408*	0.000	0.424*	0.000	0.463*	0.000	0.467*	0.000
Impact index								
*n*	324		325		325		326	
Pearson's	0.572*	0.000	0.553*	0.000	0.621*	0.000	0.753*	0.000
Spearman's	0.693*	0.000	0.699*	0.000	0.725*	0.000	0.762*	0.000

HFSS, Heart Failure Symptom Survey; MLHFQ, Minnesota Living with Heart Failure Questionnaire; *n*, number of participants.

* Adjusted significance (two‐tailed) threshold using Bonferroni's correction is *P* < 0.006.

## Discussion

**Figure 3:**
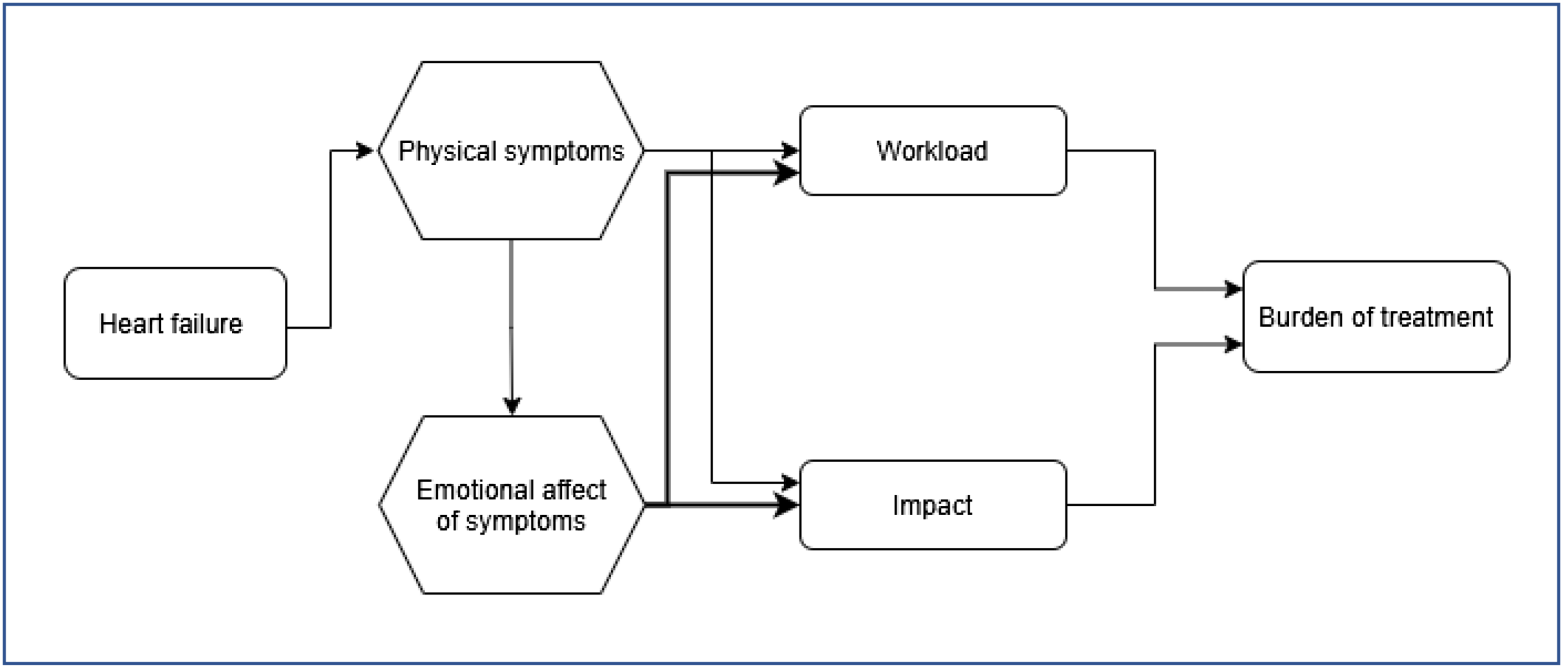
Interaction of chronic heart failure symptoms with burden of treatment. Thicker line represents strong statistical association between these factors as seen in the results.

Our survey demonstrates a positive association between symptoms (measured by HFSS and MLHFQ) and BoT (measured by PETS) in CHF patients (*Figure* *3*). As symptom burden increases, there is a positive stepwise increase in workload and impact and thereby BoT. Our exploration into the association between symptoms and BoT, measured by these questionnaires, revealed that symptoms and workload index appear to have a weak association, while symptoms and impact index have a moderate to strong association. This shows that symptoms may have a more direct influence on BoT than previously thought.

With respect to 12 of the 14 symptoms, included in the HFSS, many of our participants denied symptom burden. Symptoms of shortness of breath and fatigue had the highest burden and were more commonly reported. Our population reported lower HFSS burden score in comparison with others.^28^, ^29^, ^30^ However, it was similar to HFFS score reported by Graven *et al*.,^15^ which may be a reflection in differences in sample characteristics, difference in CHF care provision, or due to recent improvements in pharmaceutical CHF treatments. However, MLFHQ sub‐scores (physical and emotional) were similar to those reported elsewhere.^31^, ^32^, ^33^, ^34^ The lower symptom score may reflect this population being on optimal treatments for CHF; for example, 41% were receiving sacubitril valsartan. Assessing patient experience of symptoms in CHF is challenging without agreed gold standard tool.^18^

Despite a low median symptom burden score (HFSS), participants reported moderate mean levels of burden in PETS domains of *exercise/physical therapy*, *diet*, *difficulty with heartcare services*, and *physical and mental fatigue*. In our population, PETS individual domain scores were between those reported in a multi‐morbid population^22^ and those reported in a Norwegian CHF population.^35^ At least 50% of our participants denied healthcare professionals having advised them about specific diet or exercise recommendations. Both of which are considered important factors in CHF self‐care^36^ and are often associated with poor self‐care practices potentially contributing to readmissions.^37^ The BoT dimension of *difficulty with healthcare services* had the third highest reported burden, which has parallels with previous work highlighting that the complexity of healthcare service provision can be barrier to CHF patients engaging with self‐care.^38^

Symptoms have a moderate to strong correlation with the PETS impact index. The impact index captures how much engagement with self‐care regimens interferes with the domains of *role and social activity limitations* (ability to work, family responsibility, and daily and social activities) and *physical and mental fatigue* (negative emotional and physical experience from engaging with self‐care). In CHF patients in Norway, Nordfonn *et al*.^35^ observed moderate correlations between HRQoL scores (MLHFQ total score) and the BoT dimensions (role/social activity limitations and physical/mental fatigue), which are comparable with our findings. In our work, we examined this further by looking at the MLHFQ sub‐score, which showed a strong association between emotional sub‐score of MLHFQ and the impact of self‐care (PETS impact index), further adding to Nordfonn *et al*.^14^ conclusions that psychological distress adds to BoT and impairs HRQoL.

The weak to moderate correlation between symptoms and workload index was unexpected. The workload index in PETS captures how easy or difficult patients' report their workload around the domains of *medical information* (health literacy), *medications* (medication management), *medical appointments* (arranging and attending medical appointments), and *monitoring health* (illness‐specific self‐care tasks and monitoring lifestyle recommendations). CHF has complex self‐care regimes^36^ and a clinical perception of poor patient compliance.^11^ We assumed that participants would report, on average, higher workloads with associated high symptom burden. Raising the question, if the work is easy, then why are CHF patients thought to be not doing the work? Our results are contrary to others who report on the heavy self‐care burden and impact of symptoms.^1^ However, key elements of CHF self‐care workload (PETS domains: *exercise/physical therapy*, *diet*, and *difficulties with healthcare services*) are not included in PETS workload index. Our population scored these elements as having higher BoT. The reporting of high burden in these domains aligns with previous findings of disjointed healthcare services contributing to patient burden^13^, ^14^ and poor compliance with diet and exercise.^39^ These results also hint that healthcare interactions might need greater consideration to better understand the CHF patient experience of BoT. In SYMPACT Phase II (qualitative study), we will ask a subset of this population, what is the work involved in managing CHF, and do symptoms influence that work, in an effort to better understand these unexpected results.

The projects' aim was to explore if symptoms of CHF are intrinsically linked to BoT; that is, if a patient has low symptom burden, then the BoT will also be low. The spread of the data (*Figure* *1*) highlights that people with no symptoms reported both low and high burden, and it was similar for people with higher symptom scores. This suggests that other factors might also be influencing the observed association between symptoms and BoT. The authors plan to explore this further in a combination of secondary analysis of the quantitative data and in the qualitative data.

### Limitations

Chronic heart failure experience was captured in a single region in England, with a low percentage of women and ethnic minorities contributing to the data set. Also, while our study includes those with HFpEF, this was a small percentage of the sample due to the delivery of CHF services locally, in that not many individuals with HFpEF currently receive regular clinical follow‐ups. This may limit its generalizability. Between 41% and 65% of the sample denied burden in 12 symptoms in the HFSS, which may indicate the need for a larger sample if you include participants who are stable and with a lower NYHA classification. Filtering out these cases raised the median HFSS burden score to 3.5, which is closer to HFSS scores previously reported. As this study was observational, we were reliant on a combination of patients self‐reporting health information and researchers examining participant medical records, leading to some variables having a high amount of missing data.

### Clinical implications

This study described how CHF patients reported increased burden in engaging with key elements of their self‐care. Healthcare interactions had some of the highest burden scores. Healthcare service providers, clinicians, and researchers need to focus strategies to improve healthcare interactions. Additionally, more needs to be performed around communicating and supporting people with CHF in lifestyle behaviours such as dietary changes and exercise.

Our work described how greater emotional affect is associated with greater impact on a patients' role and social activity limitation as well as their physical and mental fatigue associated with engagement with self‐care activities. This high personal cost of doing that work together with difficulties in healthcare interactions likely decreases engagement in self‐care regimens, suggesting that perhaps complex healthcare systems may contribute to poor engagement with self‐care regimens. Clinicians and researchers need to incorporate the consideration of BoT together with decreasing symptom burden to enable patients to live well with CHF alongside increasing adherence to their self‐care treatment plans.

We postulate that if healthcare professionals incorporated BoT into the clinical evaluation of CHF, alongside symptom burden, this will create a more individualized approach, facilitating a more specific and supportive clinical interaction by focusing on troublesome symptoms (some of which may not be included in a typical heart failure consultation) or in decreasing illness workload.

## Conclusions

Our results add to the evidence that symptoms are associated with BoT in CHF patients. Our exploratory analysis demonstrated a moderate to strong positive association of symptoms with BoT, but more work is required to better understand which interventions may reduce BoT. Symptoms such as fatigue and breathlessness are frequently experienced even though this population's CHF was well treated. Further, the emotional affect from symptoms and engagement with self‐care is strongly associated with BoT, suggesting that clinicians should consider symptoms as more than indication for disease progression or adjustments to pharmaceutical treatments.

## Authors contributions

RCA drafted this paper. RCA designed the study with support and guidance from CRM, LS, and PRK. RCA analysed the data with statistical support and guidance from VK and LS. Interpretations of the data analysis was discussed between all authors and then written up by RCA. CRM, LS, AR, VK, and PRK critically reviewed the manuscript for intellectual and clinical content. All authors approved the final version of the paper. RCA is the guarantor.

## Conflict of interest

A.R. is a National Institute for Health Research (NIHR) Senior Investigator. The views expressed in this publication are those of the author(s) and not necessarily those of the National Institute for Health Research, NHS, or the Department of Health and Social Care.

## Funding

This work was supported as a part of a fully funded Clinical Academic Doctoral Fellowship at the University of Southampton, Portsmouth Hospitals University NHS Trust, and the National Institute for Health Research (NIHR) Applied Research Collaboration (ARC) Wessex. This article is an independent research supported in part by the NIHR ARC Wessex.
